# The role of long non-coding RNAs and circular RNAs in immune evasion of influenza A virus: recent advances

**DOI:** 10.3389/fcimb.2025.1708828

**Published:** 2025-11-21

**Authors:** Yanghua Ju, Yanchun Li, Chenxu Zhou, Xiuhua Yu

**Affiliations:** Department of Pediatric Respiration, Children’s Medical Center, The First Hospital of Jilin University, Changchun, Jilin, China

**Keywords:** long non-coding RNA, circular RNA, influenza A virus, immune evasion, antiviral response

## Abstract

The capacity of influenza A virus (IAV) to circumvent immune defenses in hosts renders it a persistent major peril to worldwide public health. Investigations conducted lately underscore the vital functions played by circular RNAs (circRNAs) and long non-coding RNAs (lncRNAs) during interactions between viruses and their hosts. This review summarizes current understanding of how lncRNAs and circRNAs participate in IAV immune evasion by regulating antiviral signaling pathways, interfering with interferon responses, modulating inflammatory cytokine production and cell metabolism, and affecting viral replication. This review examines molecular actions exhibited by particular lncRNAs and circRNAs within such interactions, assesses their suitability for therapeutic targets and diagnostic biomarkers, and outlines avenues for subsequent investigations across this fast-developing domain.

## 1 Introduction

A negative-sense single-stranded RNA virus, termed influenza A virus (IAV), induces sporadic pandemics alongside annual outbreaks, leading to considerable disease and death rates across the globe (Uyeki et al., 2022). Despite extensive research and vaccination efforts, IAV continues to pose major public health challenges, largely due to its remarkable capacity for immune evasion through antigenic drift and shift, as well as various molecular mechanisms that counteract host defense systems (Krammer et al., 2018).

In viral infections, non-coding RNAs (ncRNAs), particularly long non-coding RNAs (lncRNAs, >200 nucleotides) and circular RNAs (circRNAs), which are characterized by a covalently closed loop lacking 5’ caps or 3’ poly (A) tails, play crucial roles as modulators of gene expression and cellular functions (Liu and Ding, 2017; Liu et al., 2025). Involvement of these RNA forms extends to diverse biological pathways, featuring post-transcriptional modifications, chromatin remodeling, and transcriptional regulation (Wang and Cen, 2020; Zhang et al., 2025). Accumulating findings highlight the substantial impacts from circRNAs and lncRNAs of host origin or virus production on exchanges linking host immune responses to multiple viruses (Saayman et al., 2014; van Solingen et al., 2022; Min et al., 2023; Wang et al., 2024; Yu et al., 2024; Cao et al., 2025). Investigations advancing over time demonstrate how viruses seize control of host lncRNAs and circRNAs to enable infection or replication by them (Qiu et al., 2023; Sun et al., 2025).

LncRNAs and circRNAs have emerged as key regulators of antiviral innate immunity against IAV (Wang and Cen, 2020; Sajjad et al., 2021), whereas their roles in the adaptive immune response to IAV infection remain largely unexplored. The first line of host defense against viral invasion, antiviral innate immune responses, roughly comprises the following key steps: the detection of IAV infection and the activation of antiviral responses, which depend significantly on pattern recognition receptors (PRRs), encompassing Toll-like receptors (TLRs) and retinoic acid-inducible gene I (RIG-I)-like receptors (RLRs) (Yoneyama et al., 2004; Akira et al., 2006). Initiation of the interferon-mediated antiviral innate immune response, serving as the principal host safeguard against viral incursion, commences upon detecting pathogen- associated molecular patterns (PAMPs) like viral nucleic acids; this promptly activates subsequent signaling cascades (Janeway and Medzhitov, 2002). Transcription factors including NF-κB (nuclear factor kappa B) and interferon regulatory factor (IRF) facilitate the transcription of interferon (IFN) alongside antiviral cytokines (Iwanaszko and Kimmel, 2015), with the JAK-STAT (Janus protein tyrosine kinase-signal transducer and the activator of transcription) pathway subsequently inducing these elements to generate numerous IFN-stimulated genes (ISGs) that either directly counteract viral intrusion or engage adaptive immune mechanisms for combating such intrusion (Ivashkiv and Donlin, 2014).

This review highlights recent advances in understanding the roles of lncRNAs and circRNAs in mediating immune evasion by IAV. We provide a systematic analysis of their involvement in regulating PRR signaling, IFN responses, inflammatory cytokine production, cellular metabolism, and viral replication. Furthermore, we explore the potential applications of these insights for the development of novel diagnostic and therapeutic strategies against IAV infection (**Table 1**).

**Table 1:** Differential expression and acting target of lncRNA and circRNA that promote viral immune evasion after IAV infection.

ncRNA	Influenza virus strains	Induced expression	Mechanism	References
LncNSPL	H1N1,H3N2	up	Restricts the TRIM25- mediated K63-linked RIG-I ubiquitination	(Jiang et al., 2022)
Lnc-Lsm3b	H1N1	up	Locks RIG-I in an inactive conformation; impairs TRIM25-mediated K63-linked ubiquitination of RIG-I	(Jiang et al., 2018)
Lnc-MxA	H1N1	up	Forms RNA-DNA triplexes with the IFN-βpromoter	(Li et al., 2019)
LncRNA THRIL	H1N1,H9N2	down	Blocks IRF3 phosphorylation	(Chen et al., 2025)
LncRNA GAPLINC	H1N1,H9N2	up	ATG7 and GAPLINC may form a complex with IRF3, thereby suppressing the activation of IRF3	(Chen et al., 2024)
LncRNA USP30-AS1	H1N1,H3N2	up	Protects the protein stability of PHB1 and enhances its function	(Yu et al., 2025)
LncRNA TSPOAP1- AS1	H1N1	up	Negatively modulates Ifnb1 transcription and ISRE activation	(Wang et al., 2022)
LncRNA LUCAT1	IAV	up	Alters STAT1 function by reducing STAT1 binding at ISGs, restricts JAK-STAT signaling	(Agarwal et al., 2020)
LncRNA NRAV	H1N1	down	Reduces MxA and IFITM3, via activating H3K4me3 and repressing H3K27me3, interacts with ZONAB to affect the transcription of MxA.	(Ouyang et al., 2014)
Lnc-AROD	H1N1,H9N2	down	Binds to miR-324-5p and reduces its inhibitory effect on CUEDC2	(Zhang et al., 2023)
Lnc-Cxcl2 Lnc-CXCL2-4-1	IAV	up	Binds to and maintains the repressed chromatin state of Cxcl2 promoter through La in Cis. CXCL2-4–1 inhibits the expression of multiple cytokines by binding La.	(Liu et al., 2021)
LncRNA PCBP1-AS1	H1N1	up	Encodes PESP, which promotes autophagy by upregulating the transcription of ATG7	(Chi et al., 2024)
LncRNA ACOD1	H1N1	up	Combines with GOT2, improves the catalytic activity of the enzyme, enhances the key metabolites	(Wang et al., 2017)
LncRNA PAAN	H1N1, H3N2	up	Promotes the assembly of viral RNA polymerase via interaction with the PA protein	(Wang et al., 2018)
LncRNA IPAN	H1N1	up	Protects PB1, regulates the interaction between PB1 and RIG-I or TRIM25.	(Wang et al., 2019; Sun et al., 2025)
Lnc-ALOX12	H1N1	up	Supports the nuclear import of PB2	(Wang et al., 2025)
LncRNA PSMB8-AS1	H1N1	up	Uncertain	(More et al., 2019)
LncRNA VIN	H1N1, H3N2, H7N7	up	Uncertain	(Winterling et al., 2014)
CircMerTK	H1N1,H9N2	up	Decreases expression of critical ISGs, may sponge miR-125a-3p	(Qiu et al., 2023)
CircRNA_0050463	H1N1,H3N2	up	Binds with miR-33b-5p, promotes EEF1A1 expression	(Shi et al., 2021)
CircRNA GATAD2A	H1N1	up	Suppresses VPS34-dependent autophagy	(Yu et al., 2019)

## 2 Overview of lncRNAs and circRNAs in Viral Infections

### 2.1 Biogenesis and functions of lncRNAs

**Figure 1:**
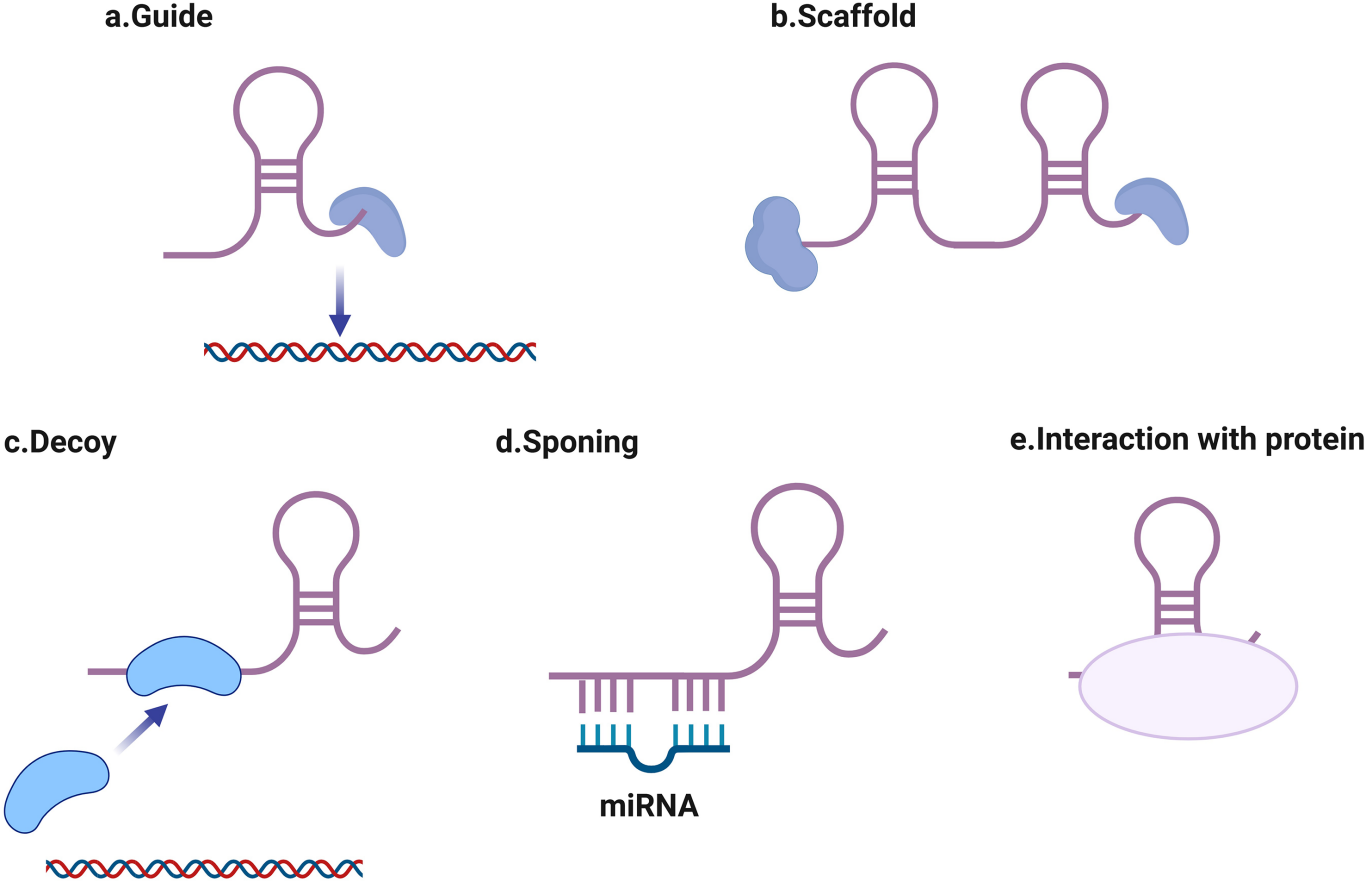
Major proposed working mechanisms of long non-coding RNAs (lncRNAs). **(a)** guiding ribonucleoprotein complexes to specific genomic loci, **(b)** acting as molecular scaffolds for protein complexes, **(c)** operating as molecular decoys to sequester proteins or miRNAs, **(d)** serving as competing endogenous RNAs (ceRNAs) that sponge miRNAs, **(e)** modulating protein activity through direct interactions.

RNA polymerase II transcribes lncRNAs, which frequently exhibit splicing and polyadenylation processes comparable to those in mRNAs; nevertheless, such molecules display negligible potential for protein encoding (Wang et al., 2024). Depending on their positions within the genome compared to protein-coding genes, these transcripts fall into categories including intergenic, intronic, bidirectional, antisense, or sense lncRNAs (Rinn and Chang, 2012; St Laurent et al., 2015). Functional mechanisms of lncRNAs include (**Figure 1**): guiding ribonucleoprotein complexes to specific genomic loci, acting as molecular scaffolds for protein complexes, operating as molecular decoys to sequester proteins or miRNAs (Balas and Johnson, 2018). Serving as competing endogenous RNAs (ceRNAs) that sponge miRNAs (Chai et al., 2018; Xu et al., 2021; Kong et al., 2024), modulating protein activity through direct interactions (Jiang et al., 2022). In viral infections, lncRNAs have been shown to regulate viral entry, replication, and host immune responses.

### 2.2 Characteristics and functions of circRNAs

**Figure 2:**
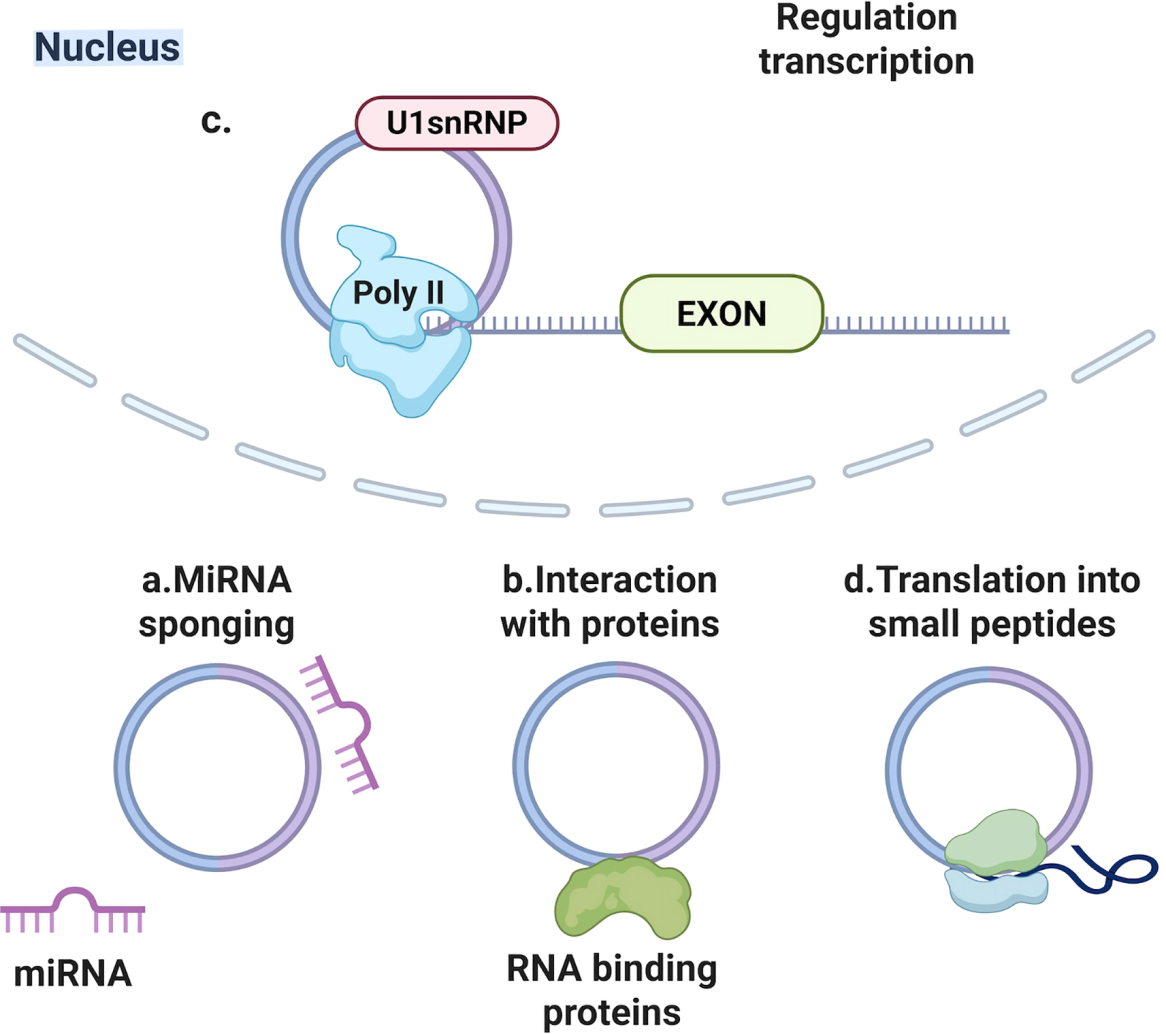
Main biological functions of circular non-coding RNAs (circRNAs). **(a)** miRNA sponging through multiple binding sites, **(b)** interaction with RNA-binding proteins, **(c)** regulation of parental gene transcription, **(d)** potential translation into small peptides.

Back-splicing yields covalently closed loop structures termed circRNAs, thereby endowing them with resistance against exonuclease-mediated degradation (Xin et al., 2021). Such transcripts manifest considerable abundance and evolutionary conservation, alongside expression profiles characteristically confined to distinct tissues or developmental phases (Wu et al., 2022). Major functions of circRNAs include (**Figure 2**): miRNA sponging through multiple binding sites, interaction with RNA-binding proteins (Hansen et al., 2013; Memczak et al., 2013; Zhang et al., 2020), regulation of parental gene transcription (Li et al., 2015), potential translation into small peptides (Legnini et al., 2017; Pamudurti et al., 2017; Yang et al., 2017). During viral infections, circRNAs can influence viral life cycles and host responses.

### 2.3 Detection and profiling methods

Recent technological advancements have enabled comprehensive detection and analysis of lncRNAs and circRNAs during IAV infection. These methods include Nanopore sequencing for full-length transcript identification (Wang et al., 2024), single-cell RNA-seq for cell-type-specific expression assessment, RNase R treatment-based protocols for circRNA sequencing (Yost et al., 2019), and high-throughput RNA-seq with ribosomal RNA depletion. Strategies for modulating lncRNA and circRNA expression primarily involve three approaches: RNA interference (RNAi) (Lennox and Behlke, 2016), antisense oligonucleotides (ASOs) (Gagnon and Corey, 2019), and RNA-specific CRISPR effectors. The latter category encompasses CRISPR-mediated gene knock-in (CRISPR-KI) and knockout (CRISPR-KO) (Petermann et al., 2019), CRISPR-mediated transcriptional activation or repression (CRISPRi/a), and CRISPR-mediated RNA silencing (Abudayyeh et al., 2017; Cox et al., 2017; Konermann et al., 2018). CRISPR/Cas13 effectors offer programmability for applications (Montagud-Martínez et al., 2024), beyond knockdown, such as gain-of-function studies using catalytically dead Cas13d (dCas13d) (Zhang et al., 2021). Furthermore, CRISPR-display facilitates fusion of ncRNA sequences to guide RNAs, enabling targeted delivery of ncRNA transcripts to specific genomic loci via CRISPR/dCas9 (Shechner et al., 2015). At the DNA level, CRISPR/Cas9 and related genome-editing tools can remove promoters, splice sites, exons, or introduce premature termination signals to regulate ncRNA expression (Horlbeck et al., 2020). Computational prediction algorithms, such as LncRNADisease v2.0 (Sheng et al., 2023), complement these experimental techniques. Collectively, these methods have uncovered dynamic alterations in lncRNA and circRNA expression profiles during IAV infection, yielding insights into their contributions to viral pathogenesis and immune modulation.

## 3 LncRNAs in IAV immune evasion

### 3.1 Regulation of PRR signaling pathways

Various lncRNAs have emerged as regulators influencing these signaling cascades:

Direct association occurs between lncNSPL (NS1-promoted lncRNA) and RIG-I, which hinders the binding of RIG-I to E3 ligase tripartite interaction motif 25 (TRIM25); consequently, this diminishes TRIM25-dependent K63-linked ubiquitination of RIG-I while suppressing the generation of subsequent antiviral factors amid the advanced phase of IAV infection. Restriction of TRIM25-dependent RIG-I K63-linked ubiquitination by lncNSPL enhances both immune evasion and IAV replication (Jiang et al., 2022). Induced by interferon, lnc-Lsm3b represents a lncRNA that rivals viral RNAs in attaching to monomeric RIG-I, leading to a feedback reduction in the innate immune role of RIG-I throughout the latter period of infection within murine macrophages. From a mechanistic perspective, activation and subsequent signaling of RIG-I are obstructed as lnc-Lsm3b secures it in a non-functional state, thereby terminating the output of type I interferons. Additionally, heightened expression of this lncRNA within L929 cells aggravates the interference with TRIM25-dependent K63-linked ubiquitination of RIG-I under conditions of viral infection (Jiang et al., 2018).

### 3.2 Interference with interferon responses

Accumulating data suggest that numerous lncRNAs modulate diverse components within innate immune responses driven by interferon:

IAV replication receives promotion from lnc-MxA (myxovirus resistance protein 1). Triplex structures comprising RNA and DNA arise when lnc-MxA interacts with the IFN-β promoter, thereby disrupting attachment of NF-κB and IRF3 to it; as a result, transcription of IFN-β gets curtailed, along with the ensuing signaling cascade (Li et al., 2019). THRIL (the TNF-α and hnRNPL-related immunoregulatory lincRNA), upon elevation, markedly boosts IAV propagation, in contrast to its knockdown, which substantially curbs viral proliferation. Phosphorylation of IRF3 receives blockade by THRIL, which in turn diminishes interferon output that encompasses select essential ISGs, thereby exerting suppressive control over host innate immunity. Expression of THRIL undergoes notable downregulation after IAV exposure in A549 cells (Chen et al., 2025). In ATG7-deficient cells, heightened levels of GAPLINC (gastric adenocarcinoma predictive long intergenic noncoding RNA) counteract the amplified IRF3 activation stemming from ATG7 absence, whereas GAPLINC reduction in ATG7-elevated cells mitigates the suppressive action of augmented ATG7 against IFN synthesis. LncRNA GAPLINC enables ATG7 to bolster IAV proliferation by dampening IRF3 phosphorylation followed by IFNs expression, operating beyond autophagy involvement. Formation of a complex uniting ATG7, GAPLINC, and IRF3 likely occurs during IAV infection, impeding IRF3 activation (Chen et al., 2024).

Influenza A virus (IAV) co-opts USP30-AS1 (USP30 antisense RNA1) to support its propagation. Induction of USP30-AS1 by IAV proceeds through the JAK-STAT pathway. Interaction of USP30-AS1 with prohibitin 1 (PHB1) occurs directly, influencing both its functional capacity and stability as a protein. PHB1 becomes sequestered from the E3 ubiquitin ligase tripartite motif containing 21 (TRIM21) due to USP30-AS1 binding, which safeguards PHB1 against degradation. Furthermore, USP30-AS1 functions as a scaffold molecule that bolsters the association between PHB1 and IRF3, ultimately blocking IRF3 translocation into the nucleus (Yu et al., 2025). Upregulation of the host lncRNA TSPOAP1-AS1 (translocator protein associated protein1-antisense RNA1) proves substantial in A549 cells subjected to IAV infection or poly (I:C) challenge. Control over TSPOAP1-AS1 elevation during IAV infection resides with the NF-κB pathway. Suppression by TSPOAP1-AS1 targets IAV-triggered Ifnb1 transcription, interferon-sensitive response element (ISRE) engagement, plus expression levels of subsequent interferon-stimulated genes (Wang et al., 2022). Removal of LUCAT1 (lung cancer associated transcript 1) triggers excessive upregulation of ISGs alongside proinflammatory cytokines within the THP-1 human monocytic line and primary human dendritic cells (DCs) exposed to LPS. In THP-1 cells, LUCAT1 elevation correspondingly weakens the IFN-α/β response elicited by stimuli. STAT1 colocalizes with LUCAT1 in nuclear compartments, where it disrupts STAT1 activity via diminished binding to ISG loci, thereby tuning their transcriptional output; such actions constrain JAK-STAT activity and facilitate immune equilibrium restoration following early viral inflammatory bursts (Agarwal et al., 2020). Viral replication escalates with NRAV (Negative Regulator of AntiViral response) overexpression, whereas NRAV depletion produces the reverse impact. Key ISGs like MxA and interferon induced transmembrane protein 3 (IFITM3) experience lowered expression mediated by NRAV through alterations in histone marks on target loci—namely, the activating H3K4me3 alongside the silencing H3K27me3. Interaction of NRAV with the transcription factor zonula occludens-1 nucleic acid-binding protein (ZONAB) occurs selectively to influence MxA transcriptional governance. During assaults by diverse viruses such as IAV, NRAV displays sharp downregulation (Ouyang et al., 2014). Downregulation characterizes lnc-AROD amid RNA virus infections. As a competing endogenous RNA, lnc-AROD sequesters miR-324-5p to counteract its suppressive action on CUEDC2 (a CUE-domain-containing protein that negatively regulates the JAK-STAT pathway to induce type I and type III interferons), consequently weakening innate immune activity and aiding IAV proliferation (Zhang et al., 2023).

### 3.3 Modulation of inflammatory responses

Severe influenza manifests dysregulated inflammation as a prominent feature, wherein lncRNAs engage in anti-inflammatory as well as pro-inflammatory mechanisms:

Association of the nucleus-localized lnc-Cxcl2 (C-X-C motif chemokine ligand 2) in mice with the Cxcl2 promoter occurs via Ribonucleoprotein La in cis, thereby preserving a chromatin configuration that remains repressed. Suppression by lnc-Cxcl2 targets Cxcl2 expression selectively within murine lung epithelial cells, excluding macrophages; post-influenza virus exposure, mice devoid of lnc-Cxcl2 demonstrate elevated Cxcl2 levels, augmented neutrophil infiltration, plus heightened pulmonary inflammation. Research further disclosed, however, that human lnc-CXCL2-4-1—deviating from the cis-operating murine lnc-Cxcl2—curtails expression across diverse cytokines such as interferons and chemokines through La attachment in human lung epithelial cells amid influenza virus challenge (Liu et al., 2021). Upregulation characterizes LncRNA PCBP1-AS1 (polycytosine binding protein 1 antisense1) after IAV infection; PCBP1-AS1 enhances influenza virus propagation via production of PESP, a compact protein. Induction of autophagy stems from PESP-mediated elevation in ATG7 transcription, coupled with PESP stabilization through its linkage to heat shock protein 90 alpha family class A member 1 (HSP90AA1) (Chi et al., 2024).

### 3.4 Modulating cellular metabolism

Exposure to numerous viruses, among them IAV, elicits induction of lncRNA-ACOD1 (aconitate decarboxylase 1). Augmentation of catalytic function in the metabolic enzyme glutamate- oxaloacetate transaminase (GOT2) results from its direct cytoplasmic association with lncRNA-ACOD1; consequently, this boosts output of vital metabolites essential to virus propagation and supports replication of influenza virus in A549 cells (Wang et al., 2017).

### 3.5 Viral replication and assembly

Direct modulation of IAV replication processes arises from multiple lncRNAs:

Induction of lncRNA-PAAN (PA-associated non-coding RNA) occurs during IAV infection without reliance on interferon, enabling its targeted utilization by influenza A virus for propagation support. Reduction in lncRNA-PAAN levels markedly curtails IAV proliferation by compromising viral RNA-dependent RNA polymerase (RdRp) functionality. Association between lncRNA-PAAN and the viral PA protein, a pivotal element within the IAV RNA polymerase assembly, underlies this effect, facilitating polymerase complex formation to ensure robust viral RNA production (Wang et al., 2018). Requirement for lncRNA-IPAN (Influenza virus PB1-associated long noncoding RNA) extends to IAV proliferation. Protection against degradation targets the viral polymerase basic protein 1 (PB1) via IAV-mediated hijacking of IPAN, which sustains effective viral RNA generation. Specific elevation of IPAN follows IAV exposure, bypassing IFN involvement (Wang et al., 2019). Recent investigations indicate that IPAN depletion provokes PB1 breakdown linked to viral RNA production; degradation of PB1 receives facilitation from RIG-I alongside TRIM25 through a mechanism devoid of signaling, while IPAN governs PB1 engagement with RIG-I or TRIM25 (Sun et al., 2025). Elevation marks lnc-ALOX12 (arachidonate 12-lipoxygenase) exclusively in cells harboring IAV, fostering viral replication. Insights from mechanistic analyses disclose lnc-ALOX12 linkage to the PB2 component of the viral RNA polymerase unit, amplifying PB2 binding to importin-α/β to aid PB2 nuclear translocation and thereby permit streamlined IAV RNA synthesis. Crucially, lnc-ALOX12 aids PB2 acclimation to host nuclear import systems amid influenza virus cross-species transfer (Wang et al., 2025).

### 3.6 Other host long non-coding RNAs regulate the replication of influenza A virus

Type I IFN alongside diverse influenza virus variants elicits upregulation of PSMB8-AS1 (PSMB8 antisense RNA1). Suppression via CRISPR interference application curtails viral protein and mRNA quantities, coupled with impediment to the dissemination of offspring influenza virus units. Clarification is pending regarding the specific positioning and function within the nucleus for PSMB8-AS1 amid influenza virus exposure (More et al., 2019).

Upregulation of VIN (Virus inducible lincRNA) arises from vesicular stomatitis virus plus multiple IAV variants (H7N7, H3N2, H1N1). RNA interference-mediated elimination hampers synthesis of viral proteins alongside IAV proliferation, underscoring the importance of this lincRNA in enabling successful IAV infection. Localization in the nucleus for VIN implies participation in regulatory gene activities, though the precise process stays undefined (Winterling et al., 2014).

## 4 CircRNAs in IAV immune evasion

Engagements involving circRNAs and influenza virus have received attention in select investigations.

Elevation of CircMerTK, originating from pre-mRNA of myeloid-epithelial-reproductive tyrosine kinase (MerTK), occurs substantially upon IAV challenge; analogous upregulation follows exposure to diverse RNA and DNA viruses across murine and human cell cultures. Alteration in IFN-β output alongside associated downstream cascades by CircMerTK yields reduced abundance of vital ISGs including IFITM3 and MX1, correlating with amplified levels of viral NP mRNA plus protein, which in turn bolsters IAV proliferation. Sequestration of miR-125a-3p potentially proceeds through CircRNA-MerTK; yet, elucidation of the circMerTK–miR-125a-3p linkage and its contribution to innate antiviral protection remains forthcoming (Qiu et al., 2023). CircRNA_0050463 engagement with miR-33b-5p has been documented, thereby elevating eukaryotic translation elongation factor 1 alpha 1 (EEF1A1) abundance and supporting IAV propagation along the circ_0050463/miR-33b-5p/EEF1A1 route (Shi et al., 2021). Influenza virus expansion receives amplification from Circ-GATAD2A (circular RNA GATA Zinc Finger Domain Containing 2A) through attenuation of autophagy reliant on VPS34 (vacuolar protein sorting 34) in A549 cell lines—a mechanism that constrains viral proliferation (Yu et al., 2019).

## 5 Diagnostic and therapeutic potential of lncRNAs and circRNAs

LncRNAs and circRNAs serve as key regulators of pathophysiological processes in various diseases (Loganathan and Doss C, 2023). These molecules hold potential as biomarkers and therapeutic targets in conditions such as cardiovascular diseases (Stepien et al., 2018; Singh et al., 2023), autoimmune diseases (Chen et al., 2019; Wen et al., 2023), cancers (Jiang et al., 2021; Chen et al., 2022; Kristensen et al., 2022), and viral infections (Mo et al., 2021; Yang et al., 2021; Wang et al., 2023). Recent studies increasingly address this area. To date, research has primarily targeted DNA viruses for diagnostic and therapeutic applications (Hong et al., 2020; Zhang et al., 2022). In contrast, studies on respiratory RNA viruses remain limited (Yao et al., 2021). Investigations specific to influenza A virus are particularly sparse, likely due to the availability of established clinical assays and treatments. However, the pursuit of precision medicine, combined with the emergence of drug-resistant influenza strains, necessitates ongoing refinement of diagnostic and therapeutic approaches. Technological advancements further enable the transition from functional characterization of lncRNAs and circRNAs to clinical implementation. Loss-of-function strategies often provide a direct means of manipulation; thus, lncRNAs and circRNAs that promote influenza A virus immune evasion emerge as prime candidates for therapeutic intervention.

Expression of lncRNA C1RL-AS1 exhibited upregulation in peripheral blood from children with IAV pneumonia. ROC analysis indicated that C1RL-AS1 predicted IAV pneumonia with an AUC of 0.957. Knockdown of C1RL-AS1 suppressed viral replication protein production, enhanced A549 cell survival, and reduced apoptosis. These findings highlight the diagnostic and therapeutic potential of C1RL-AS1 in IAV pneumonia. The regulatory role of C1RL-AS1 operates through the “C1RL-AS1/miR-16-5p/LAMP3” axis, although the precise molecular mechanism remains incompletely understood and thus not discussed in this review (Liao et al., 2025). LncNSPL displayed elevated expression in monocytes from IAV-infected patients and enhanced viral replication during the late phase of infection. Despite lacking conservation between humans and mice, lncNSPL bound to RIG-I in mouse models, as revealed by RNA pull-down assays. Overexpression of lncNSPL in AAV-lncNSPL mice boosted IAV replication and exacerbated lung injury. Given its significantly increased in peripheral blood of IAV-infected patients, lncNSPL may serve as a candidate biomarker for IAV infection, while its inhibition could offer a therapeutic avenue to mitigate infection-associated lung damage (Jiang et al., 2022). Chemical mapping and SHAPE analysis recently delineated the secondary structure of lncRNA-PAAN both *in vitro* and in cellular contexts. Structural motifs with potential functional relevance emerged during IAV infection, guiding the design of structure-specific ASOs for lncRNA-PAAN. Several such ASOs reduced lncRNA-PAAN levels and attenuated IAV infection, supporting their utility in developing novel host lncRNA-targeted anti-IAV strategies (Ulanowska et al., 2025). Differential expression profiles revealed a substantial cohort of circRNAs in patients with IAV-induced ARDS. CircRNA Slco3a1 and Wdr33 exhibited marked dysregulation across human and mouse specimens, implying biological involvement in IAV-associated lung injury based on bioinformatics predictions (Wang et al., 2021). Such investigations remain preliminary and require expanded validation.

However, translating these findings into clinical applications encounters ongoing challenges. Most studies remain at the experimental stage, with persistent technical obstacles in lncRNA- and circRNA-based therapies, including inefficient *in vivo* delivery systems and unpredictable molecular persistence. Addressing these barriers requires precise identification of relevant RNA motif/s, effective targeting, and deeper insights into the structural and functional features of lncRNAs and circRNAs.

## 6 Summary

**Figure 3:**
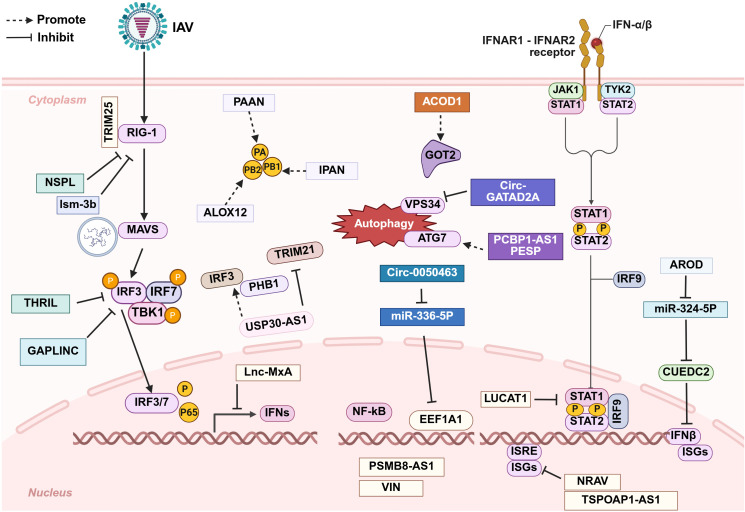
Schematic diagram of the roles that long non-coding RNAs (lncRNAs) and circular non-coding RNAs (circRNAs) play in promoting immune evasion after IAV infection. The majority of these non-coding RNAs facilitate IAV replication by modulating the host immune response at various stages, while a small subset act directly on the virus itself. Further details are provided in the main text.

Although numerous host-derived lncRNAs and circRNAs have been shown to facilitate viral immune evasion through diverse mechanisms (**Figure 3**), it is noteworthy that certain ncRNAs enhancing viral replication are downregulated during influenza virus infection. In addition, some ncRNAs attenuate inflammatory cytokine release in the late stage of infection via negative feedback, thereby mitigating immune-mediated tissue damage. This phenomenon may represent a host self-protective mechanism. To date, no influenza A virus-encoded lncRNAs or circRNAs have been identified that promote viral immune evasion.

Accumulating studies on lncRNAs and circRNAs have fundamentally advanced the understanding of IAV-host interactions and immune evasion. These ncRNAs engage in nearly all aspects of antiviral defense and viral counterstrategies, thereby shaping complex regulatory networks that ultimately influence infection outcomes. Despite remaining challenges, ongoing research on these molecules holds considerable potential for the development of innovative diagnostic and therapeutic approaches against influenza. As the field continues to evolve, the integration of ncRNA biology with classical virology is expected to drive important breakthroughs in combating influenza and other viral diseases.
